# An expedited screening platform for the discovery of anti-ageing compounds in vitro and in vivo

**DOI:** 10.1186/s13073-024-01349-w

**Published:** 2024-07-02

**Authors:** Celia Lujan, Eleanor Jane Tyler, Simone Ecker, Amy Philomena Webster, Eleanor Rachel Stead, Victoria Eugenia Martinez-Miguel, Deborah Milligan, James Charles Garbe, Martha Ruskin Stampfer, Stephan Beck, Robert Lowe, Cleo Lucinda Bishop, Ivana Bjedov

**Affiliations:** 1 UCL Cancer Institute, Paul O’Gorman Building, University College London, 72 Huntley Street London, London, WC1E 6DD UK; 2 Centre for Cell Biology and Cutaneous Research, Blizard Institute, Barts and The London Faculty of Medicine and Dentistry, Queen Mary University of London, 4 Newark Street, London, E1 2AT UK; 3 University of Exeter Medical School, Exeter, UK; 4 Max Planck Institute for Biology of Ageing, Cologne, Germany; 5 Biological Systems and Engineering Division, Lawrence Berkeley National Laboratory, Berkeley, CA USA; 6 Centre for Genomics and Child Health, Blizard Institute, Barts and The London Faculty of Medicine and Dentistry, Queen Mary University of London, 4 Newark Street, London, E1 2AT UK

**Keywords:** Senescence, CellPopAge epigenetic Clock, Drug discovery, Ageing, CpG methylation, Rapamycin

## Abstract

**Background:**

Restraining or slowing ageing hallmarks at the cellular level have been proposed as a route to increased organismal lifespan and healthspan. Consequently, there is great interest in anti-ageing drug discovery. However, this currently requires laborious and lengthy longevity analysis. Here, we present a novel screening readout for the expedited discovery of compounds that restrain ageing of cell populations in vitro and enable extension of in vivo lifespan.

**Methods:**

Using Illumina methylation arrays, we monitored DNA methylation changes accompanying long-term passaging of adult primary human cells in culture. This enabled us to develop, test, and validate the CellPopAge Clock, an epigenetic clock with underlying algorithm, unique among existing epigenetic clocks for its design to detect anti-ageing compounds in vitro. Additionally, we measured markers of senescence and performed longevity experiments in vivo in *Drosophila*, to further validate our approach to discover novel anti-ageing compounds. Finally, we bench mark our epigenetic clock with other available epigenetic clocks to consolidate its usefulness and specialisation for primary cells in culture.

**Results:**

We developed a novel epigenetic clock, the CellPopAge Clock, to accurately monitor the age of a population of adult human primary cells. We find that the CellPopAge Clock can detect decelerated passage-based ageing of human primary cells treated with rapamycin or trametinib, well-established longevity drugs. We then utilise the CellPopAge Clock as a screening tool for the identification of compounds which decelerate ageing of cell populations, uncovering novel anti-ageing drugs, torin2 and dactolisib (BEZ-235). We demonstrate that delayed epigenetic ageing in human primary cells treated with anti-ageing compounds is accompanied by a reduction in senescence and ageing biomarkers. Finally, we extend our screening platform in vivo by taking advantage of a specially formulated holidic medium for increased drug bioavailability in *Drosophila*. We show that the novel anti-ageing drugs, torin2 and dactolisib (BEZ-235), increase longevity in vivo.

**Conclusions:**

Our method expands the scope of CpG methylation profiling to accurately and rapidly detecting anti-ageing potential of drugs using human cells in vitro, and in vivo, providing a novel accelerated discovery platform to test sought after anti-ageing compounds and geroprotectors.

**Supplementary Information:**

The online version contains supplementary material available at 10.1186/s13073-024-01349-w.

## Background

Organismal ageing has been attributed to nine causal hallmarks [1] which include dysregulated nutrient sensing, increased senescent cell burden, and epigenetic alterations such as changes in DNA methylation. The latter underpins a plethora of epigenetic clocks derived from selected CpG sites which enable the accurate measurement of human age [2–5]. Consequently, slowing ageing hallmarks at the cellular level have been proposed as a route to increase organismal lifespan with the potential for increased healthspan. In support of such a strategy, there are multiple examples of genetic interventions that can extend both lifespan and healthspan in model organisms. For example, the downregulation of major cellular nutrient signalling pathways, such as glucose-sensing insulin signalling or amino acid-sensing target-of-rapamycin signalling, results in longevity and health improvement in all model organisms tested from yeast to mammals [1]. Furthermore, the selective clearance of senescent cells also extends lifespan in mice [6] and has been shown to alleviate features of an ever-growing number of age-related diseases [7, 8]. Taken together, these seminal findings show that ageing is a malleable process which has the potential to be influenced via pharmacological interventions.

Despite this promise, to date, there are only a handful of pharmacological treatments which have reliably extended lifespan in mammals. One well-studied example is rapamycin, which delays ageing in yeast, *Drosophila*, *C. elegans*, and mice [9–13]. Rapamycin has also been shown to slow age-related diseases in multiple species including humans [14]. Interestingly, there is emerging evidence that pharmacological interventions which extend lifespan may have the potential to slow epigenetic clocks. Epigenetic clocks are molecular estimators of biological age based on the DNA methylation levels of specific CpG sites across the genome [2]. For example, rapamycin slowed epigenetic ageing of a 148 CpG clock in mice [15]. However, it should be noted that daily rapamycin treatment over 2.5–3 years did not slow epigenetic ageing in marmosets, albeit the effectiveness of rapamycin-mediated lifespan extension in these experiments is yet to be reported [16].

The impact of pharmacological interventions has also been explored at the cellular level. Complementing the in vivo findings above, rapamycin can delay the onset of senescence following serial passaging in human BJ fibroblasts [17] and gingival fibroblasts [18]. More recently, rapamycin has been shown to delay senescence in neonatal keratinocytes and to slow epigenetic ageing [19] as defined by the in vivo Skin and Blood clock [20].

The utility of epigenetic clocks has also been explored at the cellular level. Early analysis using the 27 K array demonstrated highly reproducible epigenetic changes upon serial passaging of fibroblasts and mesenchymal stem cells (MSCs) [21]. We have also shown that DNA methylation changes observed during cellular senescence in adult human mammary epithelial cells can be “reset” following a cellular rejuvenation protocol [22]. As a consequence, targeted in vitro endeavours are enabling the generation of new clocks for individual ageing hallmarks or defined cellular processes which might otherwise be muffled within “bulk” in vivo datasets. CpG methylation changes can be exploited to gain insights into different aspects of biological ageing, and here we are focusing on cell population ageing as a function of proliferation with gradual accumulation of senescent cells. Using these changes as a route to identify novel drugs which restrain ageing of cell populations in vitro prior to in vivo testing has the potential to considerably accelerate the discovery of new compounds promoting healthy organismal ageing.

Here, we generate the CellPopAge Clock: a tool designed to monitor changes in the age of in vitro cell populations at the epigenetic level. The CellPopAge Clock outperforms against a panel of in vivo and in vitro epigenetic clocks in adult primary fibroblasts cultured in the absence of antibiotics. We validate the CellPopAge Clock in an independent panel of fibroblasts and demonstrate that the CellPopAge Clock has utility in other cell types. Importantly, the CellPopAge Clock can detect decelerated ageing of cell populations following pharmacological interventions with the known anti-ageing compound, rapamycin. We then use this tool to screen for novel compounds which slow epigenetic ageing of cells in vitro and uncover dactolisib (BEZ-235) and torin2, which also caused a reduction in a panel of senescence and ageing biomarkers. Finally, we explored the in vivo utility of our CellPopAge Clock screening tool and confirm that both dactolisib (BEZ-235) and torin2 are able to extend lifespan in vivo. Taken together, the CellPopAge Clock has future utility as a new screening platform for the identification of novel compounds which delay ageing of cell populations and extend in vivo lifespan.

## Methods

### Cell culture and reagents

Normal finite lifespan human mammary fibroblasts (HMFs) were obtained from reduction mammoplasty tissue of a 16-year-old individual, donor 48 by Dr Martha Stampfer (Lawrence Berkeley National Laboratory) who has all required IRB approvals to distribute these cell samples and MTA agreement set in place with Dr Cleo Bishop laboratory. Independent cultures from these cells were serially passaged from passage 9 through to passage 20 and aliquots taken upon each passage for Illumina Infinium Methylation EPIC analysis. HMFs were maintained in Dulbecco’s Modified Eagle Medium (DMEM) (Life Technologies, UK) supplemented with 10% foetal bovine serum (FBS) (Labtech.com, UK), 2 mM L-glutamine (Life Technologies, UK), and 10 μg/mL insulin from bovine pancreas (Sigma). For all passaging throughout this work, HMF cells were plated at 10,000 cells/cm^2^ in T25 cell culture flask in 5 ml of media. Media was changed every 2 days, cells were passaged every 7 days, and trypsinisation was used to detach the cells. Cells were reseeded at the same seeding density of 10,000 cells/cm^2^. Normal finite lifespan human dermal fibroblasts (HDFs) were obtained from face lift dermis following a kind donation from a 42-year-old female, anonymous healthy patient, under standard ethical practice, reference LREC No. 09/HO704/69. HDFs were grown in DMEM with 4 mM L-glutamine (Life Technologies), supplemented with 10% FBS. For all passaging throughout this work, HDF cells were plated at 7500 cells/cm^2^ in T25 cell culture flask in 5 ml of media. Media was changed every 2 days, cells were passaged every 7 days, and trypsinisation was used to detach the cells. Cells were reseeded at the same seeding density of 7500 cells/cm^2^. For drug treatments, 5 ml of appropriate drug or vehicle control was added to 5 ml of medium at each medium change and on passaging. All cells were routinely tested for mycoplasma and shown to be negative. Population doublings were calculated using the following equation: Population Doubling = (LN(Final/(Seeded/2))/0.6931) − 1.

### Immunofluorescence microscopy and high content analysis

Cells were washed in phosphate-buffered saline (PBS), fixed for 15 min with 3.7% paraformaldehyde with 5% sucrose, washed and permeabilised for 15 min using 0.1% Triton X in PBS (30 min for anti-nucleolin antibody), and then washed and blocked in 0.25% bovine serum albumin (BSA) in PBS before primary antibody incubations. Primary antibodies used were anti-IL6 (R&D Systems, 1:100; overnight 4 °C), anti-nucleolin (Santa Cruz, 1:2000, overnight room temperature), anti-p16 (Proteintech, 1:500, overnight 4 °C), anti-p21 (12D1, Cell Signalling, 1:2000, overnight 4 °C). Cells were incubated for 2 h at room temperature with the appropriate AlexaFluor-488, AlexaFluor-546, or AlexaFluor-647 conjugated antibody (1:500, Invitrogen), DAPI (1:1,000 from 1 mg/mL stock), and CellMask Orange or Deep Red (1:200,000, Invitrogen). Images were acquired using the IN Cell 2200 or 6000 automated microscope (GE), and HCA was performed using the IN Cell Developer software (GE).

### Robust Z score generation

For each of the parameters analysed, we determined the robust *Z* score relative to the negative control median value. Robust *Z* scores were generated according to the following formula: Robust *Z* score = (treatment – control)/MAD, where MAD = median(| *x* − median(*x*)|).

### Senescence-associated *beta*-galactosidase (SA-β-Gal) assay

Cells were washed in PBS, fixed for 5 min with 0.2% glutaraldehyde, washed, and incubated for 24 h at 37 °C (no CO_2_) with fresh senescence-associated beta-galactosidase (SA-β-Gal) solution: 1 mg of 5-bromo-4-chloro-3-indoyl β-D-galactosidase (X-Gal) per mL (stock = 20 mg/ml in dimethylsulfoxide)/40 mM citric acid/sodium phosphate, pH 6.0/5 mM potassium ferrocyanide/5 mM potassium ferricyanide/150 mM NaCl/2 mM MgCl_2_). Cells were stained with Hoechst 33342 (1:10,000 from 10 mg/mL stock) for 30 min. Images were acquired using the IN Cell 2200 automated microscope, and HCA was performed using the IN Cell Developer software.

### Genomic DNA extraction

For isolation of genomic DNA from primary human fibroblasts, we used QIAamp DNA micro kit (56304), and we followed manufacturers protocol, with additional washing steps with 500 μl AW2 buffer and 500 μl 80% ethanol to improve purity. DNA quantification and purity was determined by Nanodrop and QuBit. For bisulfite conversion, the EZ DNA methylation kit was used (D5001).

### Preparation of methylation array data

For each sample, 500 ng high-quality DNA was bisulphite converted using the EZ DNA methylation kit (Zymo Research), using the alternative incubation conditions recommended for use with Illumina methylation arrays. Bisulphite-converted DNA was eluted in 12 µl elution buffer. Methylation was analysed using the Infinium Human Methylation EPIC array (Illumina) using standard operating procedures at the UCL Genomics facility. The EPIC array data have been deposited into ArrayExpress at the European Bioinformatics Institute (https://www.ebi.ac.uk/arrayexpress/) under accession number E-MTAB-8327 [23].

### Pre-processing of methylation array data

DNA methylation array data was processed using the minfi package [24] within R (R Core Team, 2013). Initial QC metrics from this package were used to remove low-quality samples. Probes were filtered using a detection *p*-value cut-off > 0.01 and normalised using the Noob procedure. Cross-hybridising probes were removed from analysis based on the list published in McCartney et al. [25]. The training and test sets were pre-processed separately to obtain a fair estimate of the performance of the CellPopAge Clock.

### Estimation of sample age using existing epigenetic clocks

Following pre-processing of data, the epigenetic age of all samples was predicted using three epigenetic clocks: the Multi-Tissue clock [26], the Skin and Blood clock [20], and the PhenoAge clock [27] using the online DNA methylation calculator at http://dnamage.genetics.ucla.edu [26].

### Development of the CellPopAge Clock

The clock was built using a total of 39 samples, with six samples at each of passages 10, 12, and 14 and seven samples at each of passages 16, 18, and 20. This included both HDFs (*n* = 12) and HMFs (*n* = 27). 730,453 probes passed quality control measurements as described in the pre-processing section. A differential DNA methylation test was performed on this set of probes to identify CpGs undergoing significant DNA methylation changes with increasing cell passage. We used the linear regression approach for continuous variables implemented in minfi’s DMPFinder function [24]. This resulted in 2543 differentially methylated CpGs at a *p*-value threshold of 1 × 10^−11^ which was selected using leave one out validation. Next, we built the clock model by elastic net regression using the DNA methylation levels of the 2543 CpGs across all passages as input. We applied the glmnet function of the corresponding R package [28] setting alpha to 0.5 and determining the lambda parameter by the internal cross-validation function provided by glmnet. The elastic net regression model selected 42 CpGs as predictors of cell passage (Additional file 2: Table S1). We obtained the genomic annotation of these CpGs from Illumina’s EPIC manifest and retrieved gene functions using DAVID [29] where we selected the following sources for annotation: GOTERM_BP_DIRECT, GOTERM_CC_DIRECT, GOTERM_MF_DIRECT, ENTREZ_GENE_SUMMARY, OFFICIAL_GENE_SYMBOL, and KEGG_PATHWAY. We then tested the CellPopAge Clock on a different set of 26 samples, 22 HMFs and 4 HDFs, across the following passages: 9 (1 HMFs), 10 (2 HMFs), 11 (2 HMFs), 12 (1 HMFs), 13 (2 HMFs), 14 (2 HMFs), 15 (2 HMFs), 16 (4 HMFs and 2 HDFs), 18 (3 HMFs and 1 HDFs), and 20 (3 HMFs and 1 HDFs).

### Availability of the CellPopAge Clock

The CellPopAge Clock is available as a Jupyter Notebook in Python and can be retrieved from https://github.com/ucl-medical-genomics/CellPopAge-epigenetic-clock [30].

### Lifespan measurements

We used *white Dahomey* (*w*^*Dah*^) wild-type flies that were maintained and all experiments were conducted at 25 °C. Flies were kept on a 12-h light to 12-h dark cycle at constant humidity using standard sugar/yeast/agar (SYA) medium. For all experiments, flies were reared at standard larval density by transferring 18 μl of egg suspension into SYA bottles. Eclosing adults were collected over a 12-h period and allowed to mate for 48 h before sorting into single sexes and placed in vials containing either control or experimental drug food. Flies were routinely raised on SYA medium (standard density) and only transferred to holidic medium (15 flies per vial) for the longevity experiments. We used holidic media recipe food for all longevity assays [31]. Flies were transferred to fresh food vials every 2–3 days and scored for deaths. At least 150 flies were used for each experimental condition in all lifespan experiments.

### Western blot measurements

Whole flies or human primary cell pellets were homogenised in 2 × Laemmli loading sample buffer (100 mM Tris pH 6.8, 20% glycerol, 4% SDS; Bio-Rad) containing 50 mM DTT, protease inhibitor (cOmplete Mini EDTA-free; Roche), and phosphatase inhibitor (PhosSTOP EASYpack; Roche) cocktails. Extracts were cleared by centrifugation, and approximately 20 μg of protein extract was loaded per lane on a polyacrylamide gel. Proteins were separated and transferred to nitrocellulose membranes. The following antibodies were used at the indicated dilutions: H3 (Cell Signaling Technology; 1:2000; 4499S), pS6K (Cell Signaling Technology; 1:1000; 9206S), total S6K (Santa Cruz; 1:1000; 8418), p4EBP (Cell Signalling Technology, 1:500; 2855S), non-phospho4E-BP (Cell Signalling Technology; 1:500; 4923S), pAkt (Cell Signalling; 1:1000; 4060), pAkt (Cell Signalling; 1:1000; 4056), total Akt (Cell Signalling; 1:1000; 9272), pERK (Cell Signalling; 1:1000; 4370), total (Cell Signalling; 1:1000; 4692). Blots were developed using the ECL detection system (GE, Amersham) and analysed using FIJI software (US National Institutes of Health). We used precast TGX stain-free gels from Bio-Rad (567–8123 or 567–8124) according to the manufacturer’s instructions.

### Statistical analysis

Statistical analysis was performed using R. Log-rank tests were performed on lifespan curves.

### Online content

The CellPopAge Clock is available from GitHub at https://github.com/ucl-medical-genomics/CellPopAge-epigenetic-clock [30]. All methylation microarray data reported in this study have been deposited in the ArrayExpress (https://www.ebi.ac.uk/arrayexpress/) public repository and are accessible under accession number E-MTAB-8327 [23].

## Results

### Development of a novel CellPopAge Clock to accurately monitor epigenetic ageing in adult human primary cells in vitro

The overarching goal of this work was to establish a novel screening platform for the expedited discovery of compounds that restrain ageing of cell populations in vitro and enable extension of in vivo lifespan. Given the broad utility of epigenetic clocks [2–5], we selected DNA methylation as our in vitro readout. Therefore, our first aim was to define the epigenetic changes in a population of serially passaged adult human primary cells with the sensitivity to identify such interventions. To achieve this, we took advantage of our existing model of deep senescence in adult human mammary fibroblasts (HMFs). We had previously cultured these HMFs to deep senescence over a period of > 200 days in antibiotic free conditions and extensively characterised our model using a panel of senescence markers [32–34]. Taking these findings into consideration, we measured CpG methylation at 850,000 sites using the EPIC Array (Illumina) in HMFs from passage 10 to passage 20 at every other passage in antibiotic free conditions (Additional file 1: Fig. S1A-G). We selected this mid passage range to enable throughput of downstream compound testing.

**Fig. 1:**
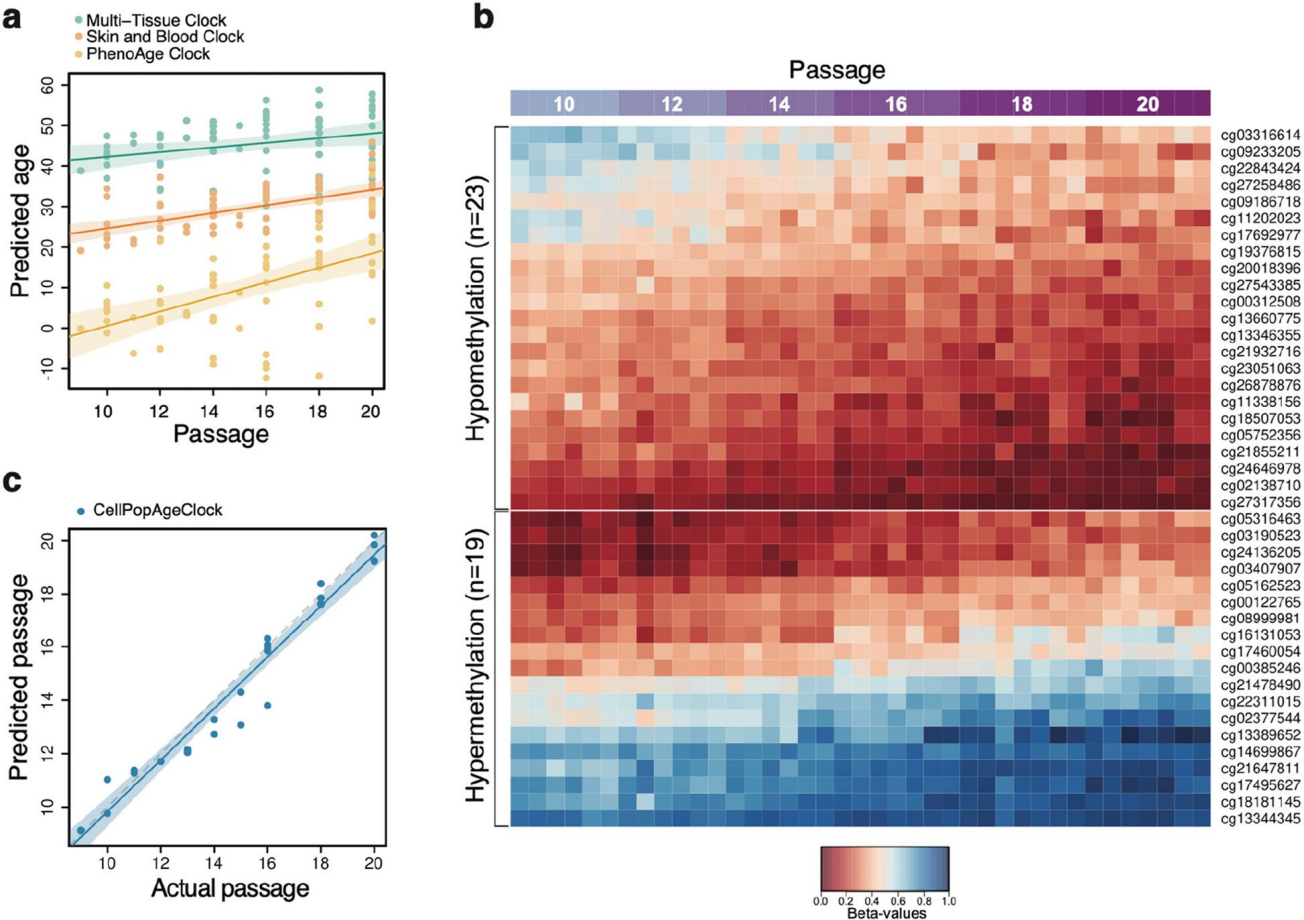
Development of the CellPopAge Clock for monitoring subtle ageing difference in cells in culture. **A** Predicted age of control samples using three existing epigenetic clocks. Predicted epigenetic age for control samples across all experiments as estimated by the Multi-Tissue clock (green), the Skin and Blood clock (orange), and the PhenoAge clock (yellow). Fitted lines are shown with 95% confidence intervals (semi-transparent). All three clocks show a trend to increase in predicted age with progressing passage; however, there is considerable variability in predictions, particularly for the PhenoAge clock. The Multi-Tissue clock consistently predicted cells to have the highest epigenetic age, whilst the PhenoAge clock consistently predicted cells to have the lowest epigenetic age, which even reached below zero for several samples at various passages. **B** Heatmap representing 23 CpG probes that undergo hypomethylation with increasing cell passage and 19 CpGs that undergo hypermethylation with increasing cell passage. These 42 CpG probes were used to develop the CellPopAge Clock. Probes that undergo hypomethylation and hypermethylation with increasing passage were separated and ordered by their methylation values per row. The mean absolute difference between passage 20 and passage 10 among clock CpGs is 0.2. **C** Testing the CellPopAge Clock on HMF and HDF samples that were not used to train the clock. The grey dashed line represents the diagonal (perfect prediction). The fitted line of the actual data is shown in blue, with a 95% confidence interval (semi-transparent). Cell passages are predicted accurately

Next, we tested the utility of the three most suitable existing epigenetic clocks. First, we asked how these clocks performed in estimating the epigenetic age of the donor (Fig. 1A). The Multi-Tissue clock [26] consistently predicted a higher epigenetic age, and at passage 10, this was 43.6 ± 1.0 years (Fig. 1A), consistent with what was recently reported [35]. This increased age estimate, compared to the age of the donor who was 16 years old, is in accordance with published data demonstrating that this epigenetic clock overestimates the age of mammary tissue samples [26]. The PhenoAge clock [27], developed to predict mortality and morbidity risks, reported the epigenetic age of the donor to be 3.5 ± 1.1 years (Fig. 1A). The most accurate age estimate, predicting the age of the donor at 23.2 ± 0.87 years, was obtained using the Skin and Blood clock [20], which is specialised for determining donor age of easily accessible human tissues and cells in culture (Fig. 1A). The Skin and Blood clock age estimate is even more accurate if the age is reverse extrapolated to passage 0, when the cells were freshly isolated, giving an age prediction of 15.11 years. We then asked if these clocks could detect weekly and monthly ageing differences occurring during serial passaging of HMFs. The Multi-Tissue clock and Skin and Blood clock showed a small increase in age with progressive passaging (from passages 10 to 20, age estimate increased from 43.6 ± 1.0 to 53.9 ± 1.7 and from 23.2 ± 0.87 to 31.6 ± 1.2 years, respectively), whilst this increase was greater for the PhenoAge clock (from 3.5 ± 1.1 to 26.6 ± 9.7 years).

This suggested that, of the tested clocks, the PhenoAge clock was the least accurate in predicting the donor’s age (Fig. 1A). The PhenoAge clock had the highest resolution in measuring the dynamic epigenetic changes that occur with cellular passaging but showed substantial variability in predictions for higher passages, which would obstruct the detection of ageing differences upon anti-ageing drug treatments. In conclusion, whilst the Skin and Blood clock [20] measures fibroblast ageing in culture, none of the existing clocks was ideally suited to accurately measure subtle anti-ageing drug potential in human primary cells in vitro, and similar comparisons have recently been reported by others [19, 35].

This prompted us to develop a new clock that, rather than predicting donor age in years, specialised in measuring methylation changes occurring during ageing of primary cells in culture and could differentiate DNA methylation states between each passage. To this end, we developed a clock using two different cell types obtained from different donors. The above-mentioned HMFs (donor age 16 years old) and human dermal fibroblasts (HDFs; donor age 42 years old) have different proliferative lifespans in vitro and different rates of DNA methylation change. Like the HMFs, the deeply senescent HDFs have been extensively characterised by a panel of senescence markers (e.g. [34]). For this study, the HDFs were serially passaged and sampled every other passage for DNA methylation analysis.

We used a total of 39 HMF and HDF samples to build the clock (see the “Methods” section). To preselect informative probes, we performed a statistical test to identify CpGs undergoing DNA methylation changes with increasing cell passage using linear regression (Additional file 1: Fig. S2A). The resulting 2543 CpGs were used to build the clock model by elastic net regression, similar to the method used by Horvath [26]. The model selected 42 predictor CpGs (“clock CpGs”), shown in Fig. 1B and Additional file 1: Fig. S2B. Of these CpGs, 23 undergo hypomethylation and 19 become hypermethylated with increasing cell passage (Fig. 1B and Additional file 1: Fig. S2B). Sixteen of the CpGs are located in intergenic regions (IGRs), whereas 14 of them are located in gene bodies and 12 in promoters, respectively (Additional file 1: Fig. S2C). Interestingly, two of the clock CpGs map to gene *GRID1*: one is located in its 3′UTR and one in the gene’s body. *GRID1* encodes a subunit of glutamate receptor channels. Several other clock CpGs are also located in genes implicated in cell receptor activity and metabolic processes, such as *LDLRAD4 and NPSR1*. Furthermore, multiple clock CpGs map to genes that play roles development as well as in the regulation of transcription and protein binding. Examples include *GGN*, *MEIS2*, *NF1*, *PROP1*, *RFX4*, *RUNX3*, and *SMARCA2* [36]. The 42 CpGs together with their detailed genomic and functional annotation are available in Additional file 2: Table S1.

After building our novel epigenetic clock to measure epigenetic ageing of cell populations in vitro, named CellPopAge Clock, we tested its performance using an entirely different set of samples (*n* = 26), consisting of 22 HMF and four HDF samples. We observed accurate prediction of passage number for both HMFs and HDFs, with a root mean square error (RMSE) of 0.37 (Fig. 1C). To compare the performance of the CellPopAge Clock with other epigenetic age predictors, we calculated Spearman’s rank correlation coefficients between the clocks’ output and actual cell passage (see Additional file 3: Table S2). The CellPopAge Clock showed the best correlation among the tested predictors, with Spearman’s rho = 0.98 and *p* < 2.2e − 16. We also tested the mitotic-like clocks EpiTOC [37, 38] and MiAge [26], for comparison. However, their correlation coefficients were negative, small, and non-significant (Spearman’s rho > − 0.3, *p* > 0.05).

Next, we tested the CellPopAge Clock in a publicly available dataset from Endicott et al. [39]. We selected this paper because the authors provided detailed information as to the culture regime for the serial passaging of different cell types from multiple donors prior to Infinium Methylation EPIC array analysis. We find that the CellPopAge Clock is able to accurately predict the passages of four independent fibroblast donors (RSME < 1, Spearman’s rho = > 0.93, *p* < 0.0001) as well as adult vascular endothelial cell, adult vascular smooth muscle cell, and neonatal foreskin keratinocyte (RSME < 1, Spearman’s rho = 0.8592, 0.7893, and 0.8901, respectively *p* < 0.0001 for all; Additional file 4: Table S3). This suggests that the CellPopAge Clock has utility across a range of different cell types in culture in detecting the age of cell populations.

Two previous publications questioned the influence of senescence on measuring cellular age in vitro [33, 34]. These papers ran the Multi-Tissue and Skin and Blood clocks on serially passaged fibroblasts which had been cultured to senescence (empty vector) or immortalised by hTERT. For both clocks, the predicted age of the hTERT immortalised cells continued to increase with increasing passage and thus these two previously published clocks were not able to uncouple “chronological age” in vitro from senescence, and the authors concluded that immortalised cells continue to epigenetically age over time.

When we ran the CellPopAge Clock on serially passaged fibroblasts with the control vector, the CellPopAge Clock was able to accurately predict the passage of these cells (RSME 0.9326, Spearman’s rho 0.9874, *p* < 0.001). However, interestingly, when these cells were serially passaged following immortalisation with hTERT, the CellPopAge Clock detected a gradual increase in the predicted age of these cells; however, it consistently underestimated the predicted passage of these cells. As a result, the CellPopAge Clock was not able to accurately predict the passage of these cells (Additional file 1: Fig. S3A; RSME 11.25 and Additional file 5: Table S4). These findings present three interesting potential conclusions: (1) sustained hTERT expression enables cells to continue to proliferate whilst potentially slowing but not stopping their rate of “epigenetic ageing”; (2) the CellPopAge Clock is indeed able to measure the epigenetic age of a population of non-immortalised cells, enabling the uncoupling of chronological age from senescence in vitro; and (3) by measuring ageing on a cell population level, the CellPopAge Clock captures changes in proliferation and senescent-related features. A caveat to these findings is that our machine learning model could be predicting later passages for the hTERT immortalised cells poorly because the CellPopAge Clock was not trained on very high passage cells.

### The CellPopAge Clock detected decelerated ageing of human primary cells treated with either mTOR, MEK/ERK, or PI3K pathway inhibitors

Having built a precise epigenetic clock that measures methylation changes during ageing of adult human primary cells in vitro, we tested if anti-ageing drug treatment of HMFs and HDFs decelerated the CellPopAge Clock. We chose an mTOR inhibitor, rapamycin, which is one of the most robust and evolutionarily conserved anti-ageing drug targets [40], which mediates its effect through downregulation of S6K and Pol III and upregulation of autophagy [9, 41]. We specifically selected a relatively low rapamycin concentration (5 nM). Treatment with this dose did not significantly affect the total number of population doubles observed over 11 weeks of culture when compared to the EtOH solvent control (Additional file 1: Fig. S1A and S1E-G) but did reduce mTOR signalling, as evidenced by decreased pS6K and p4E-BP phosphorylation (Additional file 1: Fig. S4). This setup intentionally mimicked the pro-longevity effects of rapamycin in vivo where it is well accepted that only mild nutrient sensing pathway inhibition increases lifespan and healthspan [1, 42].

**Fig. 2:**
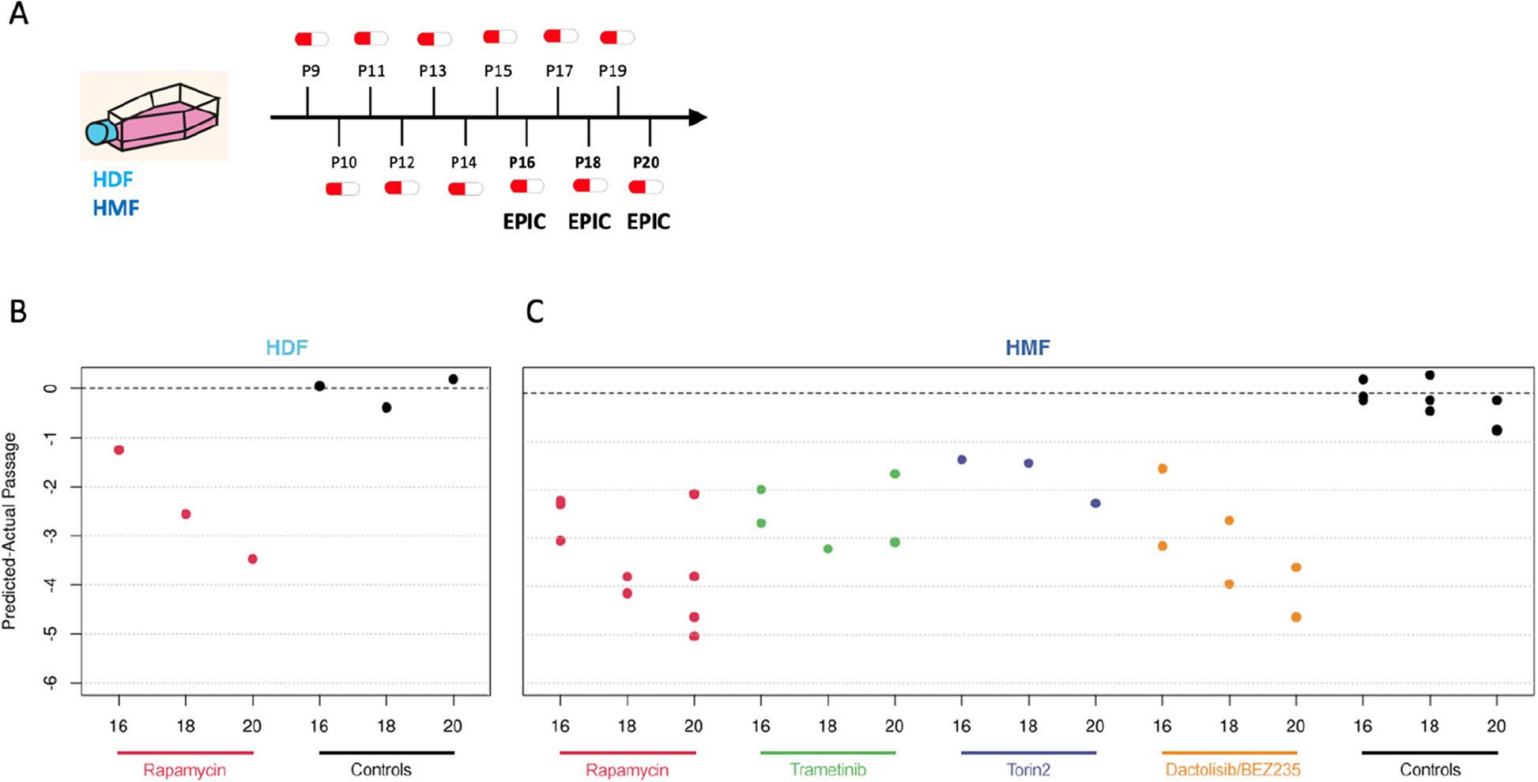
Using the CellPopAge Clock for the detection of anti-ageing drugs. **A** Schematic illustrating the experimental set-up conducted in P9 to P20 HDFs and HMFs, passaged weekly and continuously treated with either rapamycin, trametinib, torin2, or dactolisib/BEZ235, represented as a pill. Control cells were treated with vehicle, either DMSO or ethanol. **B** The CellPopAge Clock predictions of human dermal fibroblasts (HDF) and **C** human mammary fibroblasts (HMF). Represented is predicted-actual passage for passages 16, 18, and 20, showing deceleration of the CellPopAge Clock upon treatment with anti-ageing drugs rapamycin (5 nM), trametinib (0.1 nM), torin2 (5 nM), and dactolisib/BEZ235 (10 nM) and non-treated control samples (black dots)

DNA methylation profiles from HMFs and HDFs collected following 7, 9, and 11 weeks of 5 nM rapamycin treatment (passages 16, 18 and 20; Fig. 2) were analysed using the CellPopAge Clock and clearly demonstrated that rapamycin slows down methylation changes associated with ageing of cell populations. Interestingly, this clock deceleration was more pronounced upon longer treatment as shown by the gradual decrease of predicted-actual passage from passages 16 to 20. Importantly, when we compare the cumulative population doublings (CPDs) over the extended treatment period with 5 nM rapamycin (Additional file 1: Fig. S1E-G), we see no significant difference compared to the EtOH control (CPDs EtOH: 25.52 ± 1.29; 5 nM rapamycin: 24.12 ± 0.67; *p* = 0.17, *t*-test) nor do we see a significant difference in CPDs between rapamycin and EtOH controls if we perform simple linear regression analysis on passage 16 to passage 21 (*p* = 0.84, Additional file 1: Fig. S1E). Importantly, the difference between rapamycin samples and controls was pronounced when predicted passage and cumulative population doublings are compared (Additional file 1: Fig. S5A-B), with rapamycin samples estimated by the CellPopAge Clock to be four passages younger than the control. We observed a similar pattern for HMFs and HDFs (Fig. 2), suggesting that the CellPopAge Clock is applicable to different in vitro models and for cells isolated from two different tissues (breast and skin), albeit calibration is required for cells that reach senescence at different rates.

We then focused on HMFs to test another anti-ageing drug, trametinib [43], an inhibitor of the MEK/ERK signalling pathway, which was shown to extend lifespan in *Drosophila* [43]. When applied in low concentration, trametinib did not affect cell proliferation and population doublings (Additional file 1: Fig. S1B and S4), and the CellPopAge Clock analysis of trametinib treatment showed clock deceleration for all three passages tested (Fig. 2); therefore, under these conditions, the CellPopAge Clock output was solely affected by ageing of the population in vitro.

### The CellPopAge Clock as a tool for the identification of compounds which decelerates ageing of cell populations

Next, we sought to test if the CellPopAge Clock has utility for the identification of new compounds which could decelerate ageing of cell populations. We examined the effect of two other inhibitors of nutrient-sensing pathways as mutations in these pathways in model organisms represent the most evolutionary conserved anti-ageing interventions [1]. We tested dactolisib/BEZ235, a dual ATP competitive PI3K and mTOR inhibitor, for which we again optimised the dose of the treatment to obtain a reduction in signalling, as shown by pS6K downstream target 4E-BP (Additional file 1: Fig. S4), without significant proliferation impairment (Additional file 1: Fig. S1C). Dactolisib/BEZ235 slowed down the DNA methylation changes similar to rapamycin, suggesting that dactolisib/BEZ235 could be a new anti-ageing drug according to the output of the CellPopAge Clock (Fig. 2). We also tested torin2, which is a selective ATP-competitive inhibitor of the mTOR pathway that inhibits both mTORC1 and mTORC2, unlike rapamycin, which targets solely mTORC1 [44]. Owing to its more complete inhibition of the mTOR pathway, we were interested in examining its effect on ageing of cell populations, especially as the role of mTORC2 in ageing is less well established. The impact of mTORC2 inhibition on lifespan can be positive or negative depending on which of the mTORC2 downstream effectors is affected, in which tissue, and whether females or male mice are used for the experiment [45]. Some of the negative effects of mTOR pathway inhibition, such as insulin resistance and hyperlipidaemia, are attributed to the mTORC2 branch of the pathway and may arise under certain conditions of prolonged and/or high-dose rapamycin treatment [45]. Interestingly, whilst our CellPopAge Clock suggests that torin2 is indeed a novel anti-ageing drug (Fig. 2 and Additional file 1: Fig. S1D and S4), its effect on the epigenetic ageing of mammalian cell culture appears to be less pronounced than that of rapamycin. This is in line with literature suggesting that a promising strategy to improve healthy ageing is the development of inhibitors that are highly specific for mTORC1 or that target mTORC1 downstream effectors separately [45].

Next, we compared our anti-ageing drug screening results obtained by the CellPopAge Clock with analyses using Horvath’s Multi-Tissue and Skin and Blood clocks, as well as the PhenoAge clock. These clocks did not detect any significant effect of anti-ageing drug treatment (Additional file 1: Fig. S6). The Skin and Blood clock [20] was used recently to measure deceleration of ageing in primary fibroblasts [19, 35]. However, the concentration of rapamycin used in our conditions was five times lower and importantly did not affect cell proliferation, highlighting the sensitivity of our epigenetic clock to detect age-related methylation changes at very low drug concentrations. Under our conditions, the only epigenetic clock that detected gradual methylation changes from passage 10 to passage 20 was the PhenoAge clock (Additional file 1: Fig. S6). However, its output was more variable between samples and inconsistent for anti-ageing drug treatments, reporting both clock acceleration and deceleration. For instance, rapamycin-, dactolisib/BEZ235-, and torin2-treated cells appeared slightly younger compared to controls, whereas trametinib-treated cells were estimated older to some extent (Additional file 1: Fig. S6), unlike the results we obtained with our CellPopAge Clock (Fig. 2). Overall, the CellPopAge Clock that we developed here was more consistent and performed significantly better at determining the age of cells in culture and following known anti-ageing drug treatments compared to existing clocks. Our results are supportive of clocks being highly specialised for a certain task and suggests that whilst other popular epigenetic clocks perform remarkably well for determining donor’s age in years and their health status, they were not able to robustly detect delayed ageing of human primary cells induced by drug treatment over a short period of time in vitro.

### Delayed ageing in human primary cells treated with anti-ageing compounds, measured by the CellPopAge Clock, is accompanied by reduction of senescence and ageing biomarkers

**Fig. 3:**
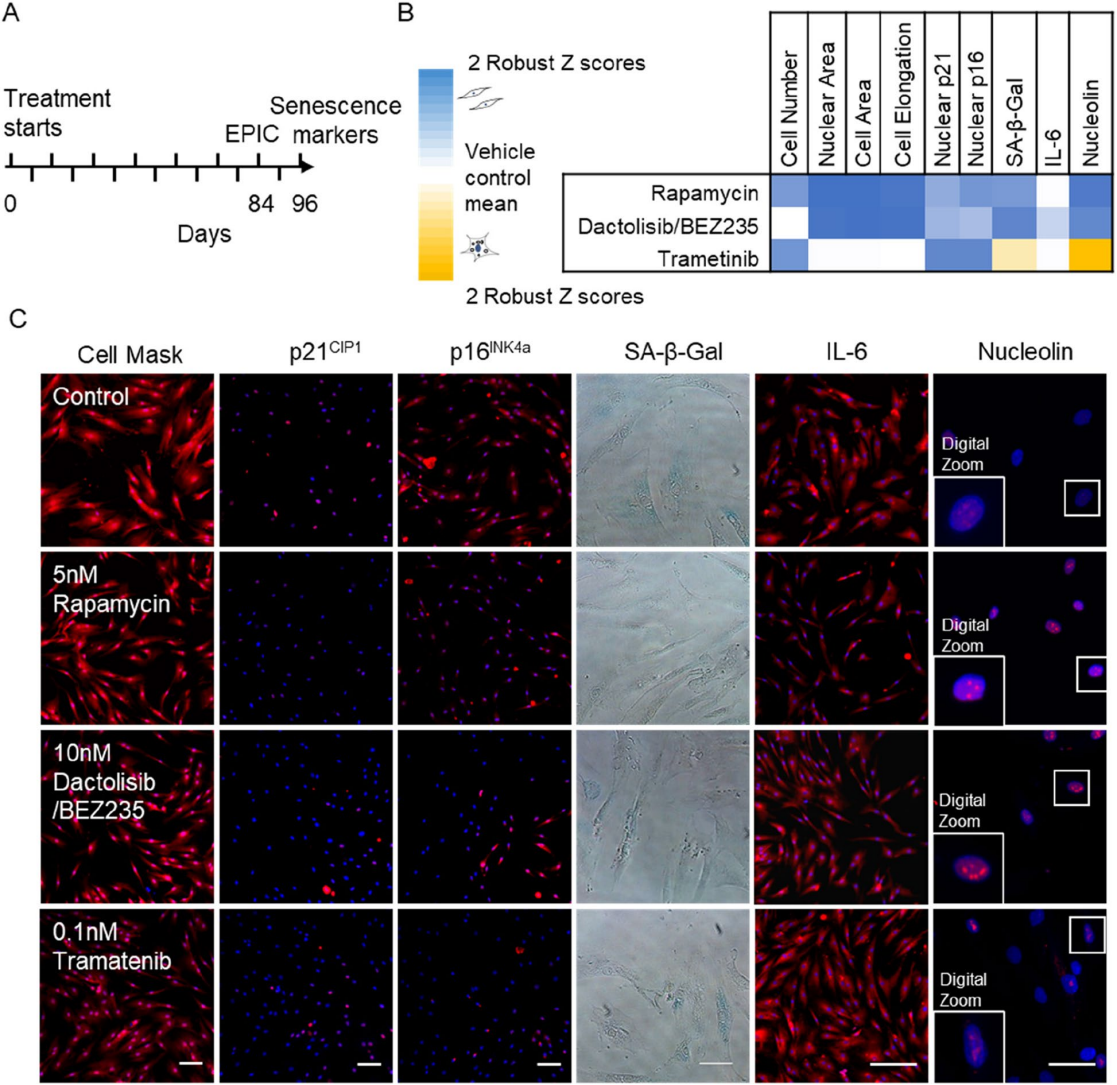
Treatment with anti-ageing drugs decreases markers of senescence. **A** Schematic illustrating the experimental set-up conducted in P10 to P22 HMFs, passaged weekly. **B** Multi-parameter analysis of senescence markers. Robust *Z* scores were calculated for a panel of measures relative to the vehicle control. Colour coding used to illustrate the number of *Z* scores of the experimental drug value from the respective vehicle control mean. Scores highlighted in blue denote a shift towards a more proliferative phenotype and scores highlighted in yellow denote a shift to a more senescent phenotype, all with a robust *Z* scores of ± 0.5. White indicates no change. *N* = 2 for all except for SA-β-gal. **C** P22 HMFs stained with DAPI (blue) and Cell Mask, p21, p16, IL-6, or nucleolin (red), or SA-β-Gal (blue) following 96-day treatment with 5 nM rapamycin, 10 nM dactolisib/BEZ235, 0.1 nM trametinib, or their respective controls. Size bar, 100 μm

Next, we assessed if drugs that decelerate the CellPopAge Clock also reduce features associated with senescence, such as morphological changes and expression of senescence and ageing biomarkers [46]. Rapamycin, dactolisib/BEZ235, and trametinib treatment slowed down morphological alteration in cells that gradually occur during ageing of cell populations, namely cell elongation, increased nuclear area and cell area, and the treated cells appearing particularly ‘youthful’ (Fig. 3). Another characteristic of senescence is increased expression of the cyclin-dependent kinase inhibitors p21^CIP1/Waf1^ and p16^INK4a^. p21^CIP1/Waf1^ triggers G1 cycle arrest upon DNA damage and can lead to senescence or apoptosis [8, 47]. Expression of p16^INK4a^, which is produced from the CDKN2A gene together with p19^ARF^ (p14^ARF^ in humans), increases exponentially during in vivo ageing and has been suggested to stabilise the senescent state [48]. p16^INK4a^ expression was also the marker of choice for senescent cell clearance leading to prolonged lifespan in mice [49].

Our results demonstrate that drugs which decelerate the CellPopAge Clock at the same time reduce expression of both nuclear p21^CIP1/Waf1^ and p16^INK4a^ compared to non-treated cells, showing their efficacy in delaying the senescence programme (Fig. 3B, C and Additional file 5: Table S5). In addition, one of the most frequently used senescent markers, senescence-associated β-galactosidase activity (SA-β-Gal), was decreased upon anti-ageing drugs treatment with rapamycin and dactolisib/BEZ235, but not in cells treated with trametinib (Fig. 3B, C). Another difference in senescent markers was observed with interleukin-6 (IL-6), which is one of the most important inflammatory cytokines and part of the senescence-associated secretory phenotype. IL-6 was significantly reduced in aged cells upon rapamycin and dactolisib/BEZ235 treatment but not in trametinib-treated cells (Fig. 3B, C). This difference possibly stems from the overactivated RAS/ERK pathway being a more prominent inducer of senescence than the overactivated mTOR/PI3K pathway [50], and hence corresponding inhibitors have different potency in inhibiting senescence. Finally, we examined the nucleolus, an organelle dedicated to rRNA production and ribosomal assembly, as it has recently emerged that maintenance of its structure, and low levels of nucleolar methyltransferase fibrillarin, is a common denominator for major anti-ageing intervention from worms to mice [51]. We observed that as a consequence of ageing, nucleoli in aged HMFs lose their defined round shape and are more diffused, with dimmer DAPI staining. For rapamycin and dactolisib/BEZ235, we observed clearly defined and ‘younger’ looking nucleoli in aged cells. However, trametinib-treated cells resembled the nucleoli of controls. In summary, a panel of the most frequently used markers for cell senescence confirmed that drugs which decelerate the CellPopAge Clock also make the cells appear more youthful. This strongly suggests that the CellPopAge Clock can be used as a robust and sensitive detector of compounds which delay ageing of cell populations in vitro.

### The two newly discovered anti-ageing compounds, torin2 and dactolisib/BEZ235, extend lifespan in vivo in *Drosophila*

**Fig. 4:**
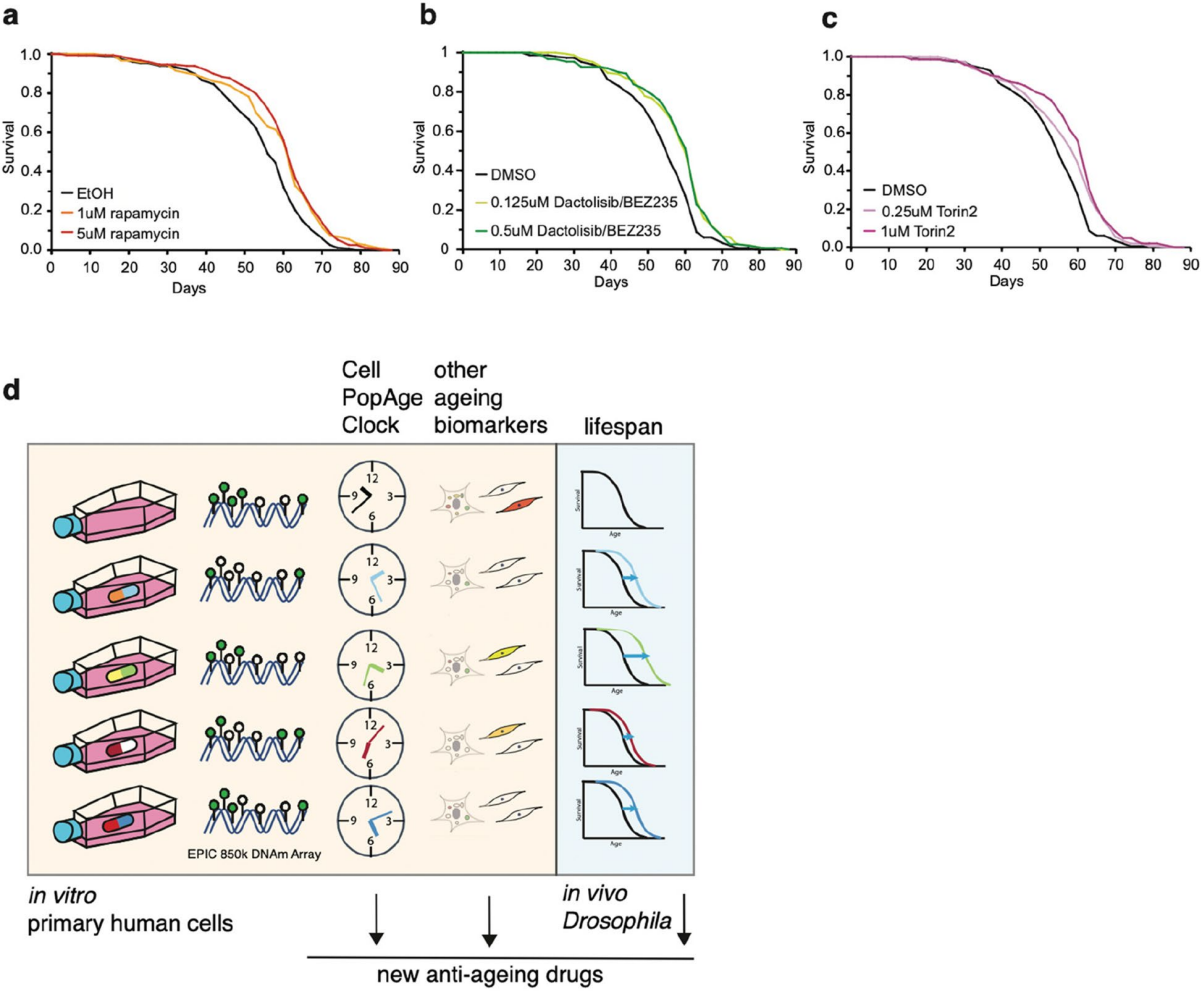
Drugs that decelerate the CellPopAge Clock extend lifespan in vivo. **A** Lifespan analysis on *w*^*Dah*^ background wild-type flies fed with holidic food containing different concentration of rapamycin or ethanol as solvent control. For each condition, 150 flies were used. **B** Lifespan analysis on *w*^*Dah*^ background wild-type flies fed with holidic food containing different concentration of dactolisib/BEZ235 or DMSO as solvent control. For each condition, 150 flies were used. **C** Lifespan analysis on *w*^*Dah*^ background wild-type flies fed with holidic food containing different concentration of torin2 or DMSO as solvent control. For each condition, 150 flies were used. **D** Schematic representation of our proposed screening platform which combines our novel CellPopAge Clock and other ageing biomarkers in vitro primary human cell, together with in vivo *Drosophila* lifespan experiments, for a detailed and robust capture of anti-ageing drug potential

Having discovered two potential novel drug treatments which delay ageing of cells in vitro using the CellPopAge Clock, we then extended our discovery platform by asking if dactolisib/BEZ235 or torin2 could influence ageing at the organismal level. This is important as tissue-specific drug toxicity, which can be missed in cell culture, is one of the major reasons for drug failure in clinical trials. To support an expedited platform, we took advantage of the model organism the fruit fly *Drosophila melanogaster*, which has well-described characteristics of cellular and organismal ageing [52]. For in vivo longevity studies, we used the outbred wild-type *w*^*Dah*^ strain which is particularly suitable for ageing studies, Drosoflipper device for fast fly transfer, and specially formulated holidic medium [31] to increase drug bioavailability compared to standard sugar-yeast-agar fly food. We used rapamycin as a positive control for these longevity experiments and showed that median lifespan extension on holidic media varied from 7 to 9% compared to ethanol solvent control, depending on 1 μM or 5 μM concentration, respectively (*p* < 0.001, log-rank test), which is comparable to published literature [53] (Fig. 4A). Importantly, both dactolisib/BEZ235 and torin2 significantly extended lifespan in *Drosophila* by 7% (*p* < 0.001, log-rank test) (Fig. 4B, C). This firmly demonstrates that drugs that decelerate the CellPopAge Clock have similarly favourable output on major anti-ageing biomarkers in vitro and extend longevity in vivo (Fig. 4D).

## Discussion

We present the CellPopAge Clock as the first robust epigenetic clock for the rapid discovery of anti-ageing drugs directly in human cells in vitro. We focused on the discovery of anti-ageing compounds which decelerated epigenetic ageing of cell populations in vitro at very low doses but which did not influence cellular proliferation. Moreover, we utilised relatively low-dose treatments of established and novel anti-ageing compounds to minimise potential off-target effects. Another relevant aspect of our design was to perform these lengthy experiments in the absence of antibiotics. Next, we coupled these anti-ageing compounds with in vivo *Drosophila* lifespan experiments as this model organism significantly shortens the discovery time compared to 3-year long mice longevity analysis. The rationale for this approach was to identify compounds with in vivo utility which uncoupled epigenetic ageing from cellular division.

Taken together, the CellPopAge Clock, based on 42 CpG sites, is a trained, tested, and validated epigenetic clock with underlying algorithm, which we believe is unique among existing epigenetic clocks as it is designed to detect compounds that restrain ageing of cell populations in vitro. Coupled with in vivo lifespan experiments in *Drosophila*, our pipeline represents a novel screening platform for the expedited discovery of anti-ageing compounds (Fig. 4D).

Initially, we benchmarked our CellPopAge Clock against other available epigenetic clocks: the Multi-Tissue clock, the PhenoAge clock, and the Skin and Blood clock. The latter was developed to measure cellular age in vitro. However, none of these epigenetic age clocks could accurately detect the effect of low-dose, short-term anti-ageing drugs on cells in vitro. Thus, our CellPopAge Clock differs from these previously published clocks in being able to measure interventions which slow both epigenetic ageing of cell populations and the accumulation of biomarkers of senescence and age across a range of different cell types. This is encouraging considering the breadth of evidence that associates cellular ageing, or senescence, with ageing and diseases of advanced age.

To provide in vivo utility for the CellPopAge Clock as a readout, we took advantage of *Drosophila* to assess the potential effect of novel anti-ageing compounds on lifespan. We selected this model as lifespan studies are comparatively short at ~ 90 days, thereby providing an expedited and relatively inexpensive in vivo model for testing potentially novel anti-ageing compounds. The development of formulated holidic medium was considered a further advantage as it provided enhanced drug bioavailability. These studies enabled us to utilise the well-established lifespan extender, rapamycin, as a benchmark, and to validate dactolisib/BEZ235 and torin2 as novel anti-ageing compounds. It should be noted that the CellPopAge Clock was not designed to measure intrinsic cell ageing or the biological age of cells in vivo.

Taken together, our results show that by using the CellPopAge Clock, cultured primary human cells can be used to measure ageing of these populations and can reliably detect anti-ageing effects upon a relatively brief, low-dose treatment. By doing so, this fast and accurate screening platform is expected to accelerate the discovery of novel preventive treatments for age-related disease, directly using human cells in vitro and in vivo lifespan experiments using *Drosophila*. Future refinements could include the use of multiplex DNA methylation-based PCR for the 42 CpG sites of the CellPopAge Clock will facilitate a screening assay for expedited drug discovery. Importantly, follow-up research should be focused on expanding our findings to different types of primary cells from donors of different ages as well as on testing further compounds. Whilst ageing itself is not a disease, potential anti-ageing drugs could be FDA approved separately for different conditions. For example, the first study to test broad-spectrum protection capacity of metformin, the TAME study, is underway [54]. In addition, it was shown that rapamycin/Everolimus pre-treatment dramatically improves flu vaccination and immune response in the elderly [55]. In mice, it also lowers the incidence of tumours [56], and it shows promising results in the field of neurodegeneration [57]. This supports the idea that targeting healthy ageing at the cellular level might have multiple beneficial outputs.

Given the wealth of information stored in our epigenome, we expect a range of biological outputs to be extracted by the CellPopAge Clock and other epigenetic clock algorithms in the future. For example, testing different compounds using the CellPopAge Clock could potentially reveal new anti-ageing pathways, currently dominated by IIS and mTOR signalling pathways. Such approaches could also help us to improve our knowledge base of not only ageing biology but of the molecular pathways underpinning the epigenetic clocks, understanding of which is limited. Our experimental setup is also suitable for nutraceutical approaches whereby dietary supplements can be rigorously tested for their effect on ageing.

At present, particularly interesting findings are emerging from the dissection of the different modules that epigenetic clocks are made of [4, 58], as well as from single cell epigenetic clock analysis [59], both of which will contribute to our mechanistic understanding of epigenetic clocks. Thus, it is interesting to speculate that a range of individual cellular hallmarks of ageing, including development and maintenance programmes, circadian rhythms, metabolism, and DNA damage and repair, might all interdependently cause gradual deterioration of CpG methylation with ageing. Focused epigenetic clock algorithms [4] through the use of in vitro systems offer a route to identifying these individual cellular fingerprints and to exploring this hypothesis. In summary, we expect our novel discovery platform to accelerate therapeutic innovation for strongly sought-after anti-ageing drugs and geroprotective strategies to improve healthy human ageing.

## Conclusions

In the present study, we have developed a novel DNA methylation clock, the CellPopAge Clock, specialised for determining age in population of primary cells in culture, for the purpose of drug screening and in the long-term developing treatments that can improve health during ageing.

### Supplementary Information

Additional file 1: Fig. S1. Drug treatment of HMF did not affect cell growth as measured by population doubling. Fig. S2. Developing the CellPopAge Clock. Fig. S3. The CellPopAge Clock predictions of Human Dermal Fibroblasts (HDF) immortalized with hTERT. Fig. S4. Western blot analysis on HMFs treated with anti-ageing drugs. Fig. S5. Comparison of cumulative population doublings following treatment with 5 nM rapamycin and control cells that were treated with ethanol as a vehicle control. Fig. S6. Predicted age of control samples and samples treated with potential anti-ageing drugs using three existing epigenetic clocks.

Additional file 2: Table S1. List of 42 CpGs, that were selected using the elastic net regression model as predictors of cell passage.

Additional file 3: Table S2. Spearman's rank correlation and RMSE of the different clocks' predictions with actual cell passage of 26 samples that were not used to build the clock.

Additional file 4: Table S3. Testing the CellPopAge Clock in a publicly available dataset from Endicott et al. [37].

Additional file 5: Table S4. The CellPopAge Clock predictions of Human Dermal Fibroblasts (HDF) immortalized with hTERT.

Additional file 6: Table S5. Expression levels of p21, p16, SA-β-Gal, IL-6 and nucleolin in treated and non-treated cells.

## Data Availability

All methylation microarray data reported in this study have been deposited in the ArrayExpress (https://www.ebi.ac.uk/arrayexpress/) public repository and are accessible under accession number E-MTAB-8327 (https://www.ebi.ac.uk/biostudies/arrayexpress/studies/E-MTAB-8327?query=E-MTAB-8327) [23]. The CellPopAge Clock is available from GitHub at https://github.com/ucl-medical-genomics/CellPopAge-epigenetic-clock [30]. Correspondence and requests for materials should be addressed to IB, CLB, RL, and SB.

## Abbreviations

3′UTR: Three prime untranslated region
4E-BP: Eukaryotic translation initiation factor 4E (eIF4E)-binding protein 1
CDKN2A: Cyclin-dependent kinase inhibitor 2A
CPD: Cumulative population doubling
DAPI: 4′,6-Diamidino-2-phenylindole
DMEM: Dulbecco’s Modified Eagle Medium
DMSO: Dimethyl sulfoxide
DTT: DL-dithiothreitol
EDTA: Ethylenediaminetetraacetic acid
EtOH: Ethanol
FBS: Foetal bovine serum
FDA: Food and Drug Administration
GGN: Gametogenetin
GRID1: Glutamate ionotropic receptor delta type subunit 1
HDFs: Human dermal fibroblasts
HMFs: Human mammary fibroblasts
hTERT: Human telomerase reverse transcriptase
IGRs: Intergenic regions
IIS: Insulin/IGF-1 signalling pathway
IL-6: Interleukin-6
LDLRAD4: Low-density lipoprotein receptor class A domain containing 4
MEIS2: Meis homeobox 2
MEK/ERK: Mitogen-activated protein kinase/extracellular signal-regulated kinase
MSCs: Mesenchymal stem cells
MTA: Material transfer agreement
mTOR: Mammalian or mechanistic target of rapamycin
NF1: Neurofibromin 1
NPSR1: Neuropeptide S receptor 1
PCR: Polymerase chain reaction
PI3K: Phosphoinositide 3-kinases
Pol III: RNA polymerase III
PROP1: PROP paired-like homeobox 1
RFX4: Regulatory factor X4
RMSE: Root mean square error
RUNX3: RUNX family transcription factor 3
S6K: Ribosomal protein S6 kinase
SA-β-Gal: Senescence-associated β-galactosidase activity
SMARCA2: SWI/SNF-related, matrix-associated, actin-dependent regulator of chromatin, subfamily A, member 2
SYA: Sugar/yeast/agar
TAME: Targeting Aging with Metformin
TOR: Target of rapamycin
