# Supplementary material for: An expedited screening platform for the discovery of anti-ageing compounds in vitro and in vivo
Source: Genome Med. 2024 Jul 2;16:85. doi: 10.1186/s13073-024-01349-w (PMC11218148; doi:10.1186/s13073-024-01349-w)
Supplement: Supplementary file 1 — Additional file 1: Fig. S1. Drug treatment of HMF did not affect cell growth as measured by population doubling. Fig. S2. Developing the CellPopAge Clock. Fig. S3. The CellPopAge Clock predictions of Human Dermal Fibroblasts (HDF) immortalized with hTERT. Fig. S4. Western blot analysis on HMFs treated with anti-ageing drugs. Fig. S5. Comparison of cumulative population doublings following treatment with 5 nM rapamycin and control cells that were treated with ethanol as a vehicle control. Fig. S6. Predicted age of control samples and samples treated with potential anti-ageing drugs using three existing epigenetic clocks. [file 13073_2024_1349_MOESM1_ESM.pdf]

SUPPLEMENTARY FIGURES

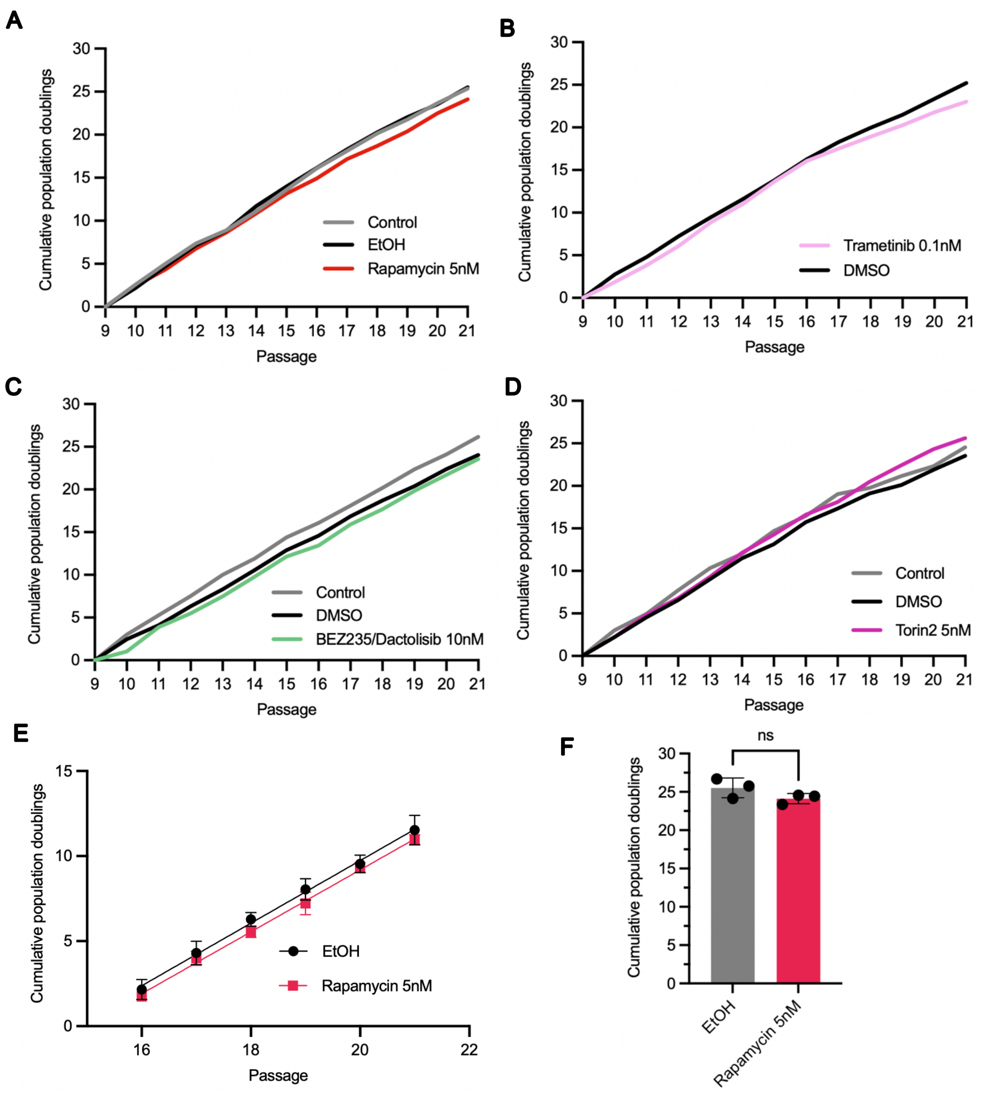

G

Passage number	Cumulative population doublings								
	**EtOH1**	**EtOH2**	**EtOH3**	**Rapamycin1**	**Rapamycin2**	**Rapamycin3**	**Control1**	**Control2**	**Control3**
**10**	1.60	2.94	2.01	2.15	2.29	2.89	2.44	2.47	2.86
**11**	3.86	5.48	4.85	3.86	4.54	4.65	4.74	5.03	5.33
**12**	6.63	7.56	7.28	6.35	6.97	6.93	7.22	7.22	7.66
**13**	7.64	9.94	8.94	7.87	9.40	8.65	8.97	8.87	8.85
**14**	10.72	13.07	11.35	9.90	11.49	11.21	10.76	10.96	11.63
**15**	13.49	15.08	13.39	12.12	13.56	13.83	13.44	13.35	14.02
**16**	14.96	17.50	15.96	14.03	15.31	15.40	15.91	15.70	16.65
**17**	17.12	19.35	18.40	16.13	17.13	18.23	18.16	17.53	18.66
**18**	19.44	21.23	20.12	17.88	19.18	19.01	19.83	19.71	20.87
**19**	21.00	22.96	22.13	19.75	21.15	20.28	21.73	21.31	22.28
**20**	22.44	24.91	23.23	21.62	23.04	22.79	23.40	23.07	24.48
**21**	24.13	26.68	25.76	23.35	24.57	24.45	25.36	24.45	26.25

Fig. S1 Drug treatment of HMF did not affect cell growth as measured by population doubling. Represented are population doubling measurements from passage 9 to passage 21 for A) Rapamycin (5nM), B) Trametinib (0.1nM), C) Dactolisib/BEZ235 (10nM), D) Torin2 (5nM) and corresponding vehicle controls. E) Cumulative population doublings for Rapamycin 5nM and EtOH vehicle control samples during the EPIC sample window (Passage 16 to Passage 20 and for a further passage to Passage 21; P=0.8394; simple linear regression). F) Comparison of cumulative population doublings from passage 10 to 21 for Rapamycin 5nM and EtOH vehicle control samples (p=0.17; t-test). G) Cumulative population doubling values for Rapamycin 5nM, EtOH vehicle control, and non-treated control samples.

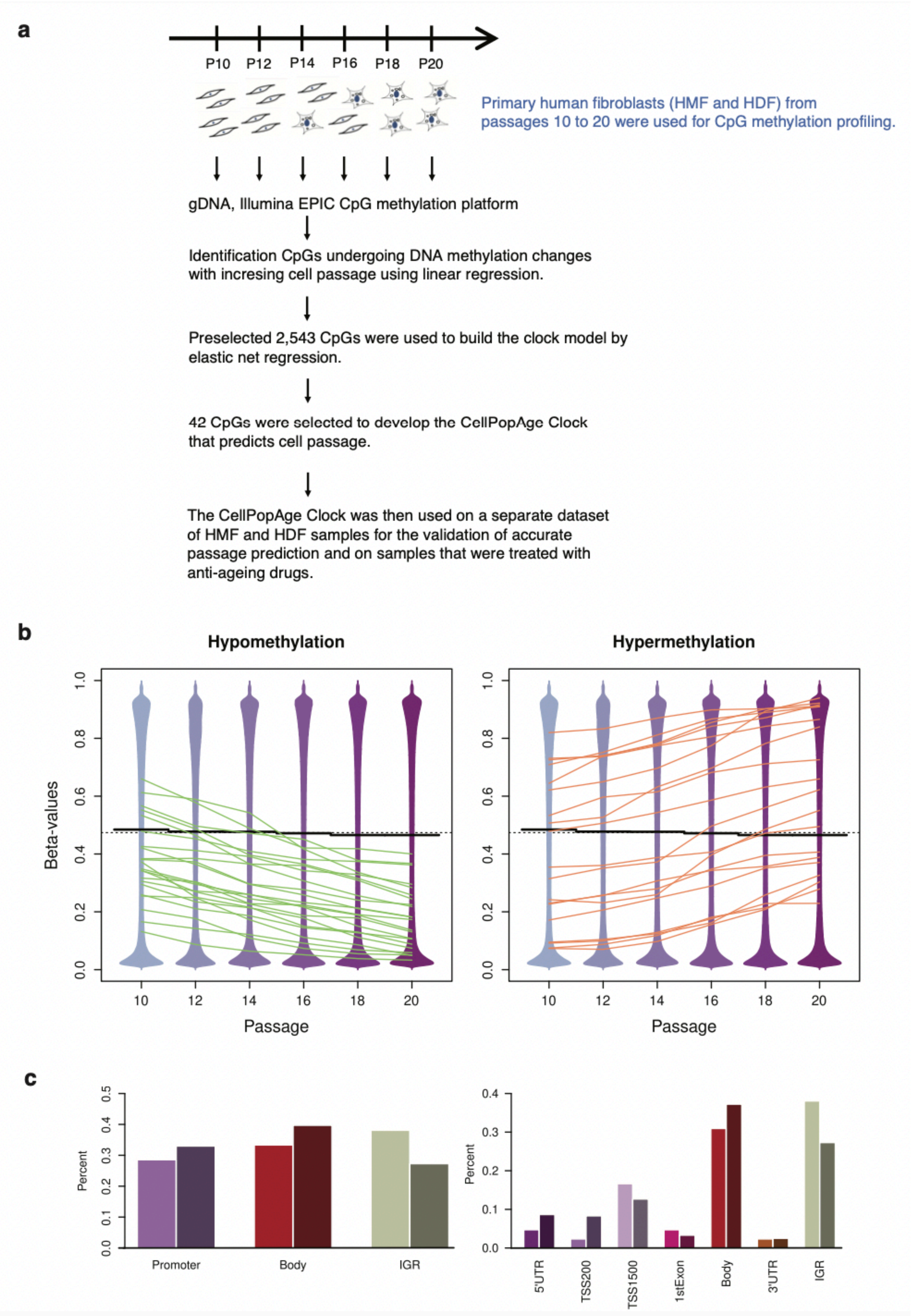
a
P10
P12
P14
P16
P18
P20
Primary human fibroblasts (HMF and HDF) from passages 10 to 20 were used for CpG methylation profiling.
gDNA, Illumina EPIC CpG methylation platform
Identification CpGs undergoing DNA methylation changes with incresing cell passage using linear regression.
Preselected 2,543 CpGs were used to build the clock model by elastic net regression.
42 CpGs were selected to develop the CellPopAge Clock that predicts cell passage.
The CellPopAge Clock was then used on a separate dataset of HMF and HDF samples for the validation of accurate passage prediction and on samples that were treated with anti-ageing drugs.
b
Hypomethylation
Hypermethylation
Beta-values
0.0
0.2
0.4
0.6
0.8
1.0
10
12
14
16
18
20
Passage
c
Percent
0.0
0.1
0.2
0.3
0.4
0.5
Promoter
Body
IGR
5'UTR
TSS200
TSS1500
1stExon
Body
3'UTR
IGR

Fig. S2 Developing the CellPopAge Clock.

A) Scheme for developing of the CellPopAge Clock. A) Primary human mammary fibroblasts and dermal fibroblasts, from passages 10 to 20, were used for CpG methylation profiling and using elastic net regression 42 CpGs were selected to develop the CellPopAge Clock that predicts cell passage.

B) The beanplots showing the overall distribution of DNA methylation values (all CpGs, n=736,001) are presented. Overall DNA methylation levels decrease with increasing cell passage. The lines show where the clock CpGs sit in the distributions (mean per passage group), and are connected across passages to see how their methylation levels change. The plots show the mean methylation changes the clock CpGs are undergoing with increasing passage, separated by those that become hypo- and hypermethylated, in the context of the global DNA methylation patterns of all CpGs.

C) Barplots showing the percentage of CpGs falling into the corresponding genomic feature. Lighter bars show the percentages of clock CpGs (x% of the 42 CpGs). Darker bars show the background distribution (all other CpGs analyzed = 735,959 CpGs). Clock CpGs fall more often into IGRs. Barplots on the left are separated into gene promoters, gene bodies and IGRs for which in the CellPopAge Clock CpGs we found 12, 14 and 16, respectively. More detailed separation of the CellPopAge Clock CpGs into 5'UTR (2 CpGs), TSS200 (1 CpG), TSS1500 (7 CpGs), 1st exon (2 CpGs), gene body (13 CpGs) and 3'UTR (1CpG) and IGR (16 CpG).

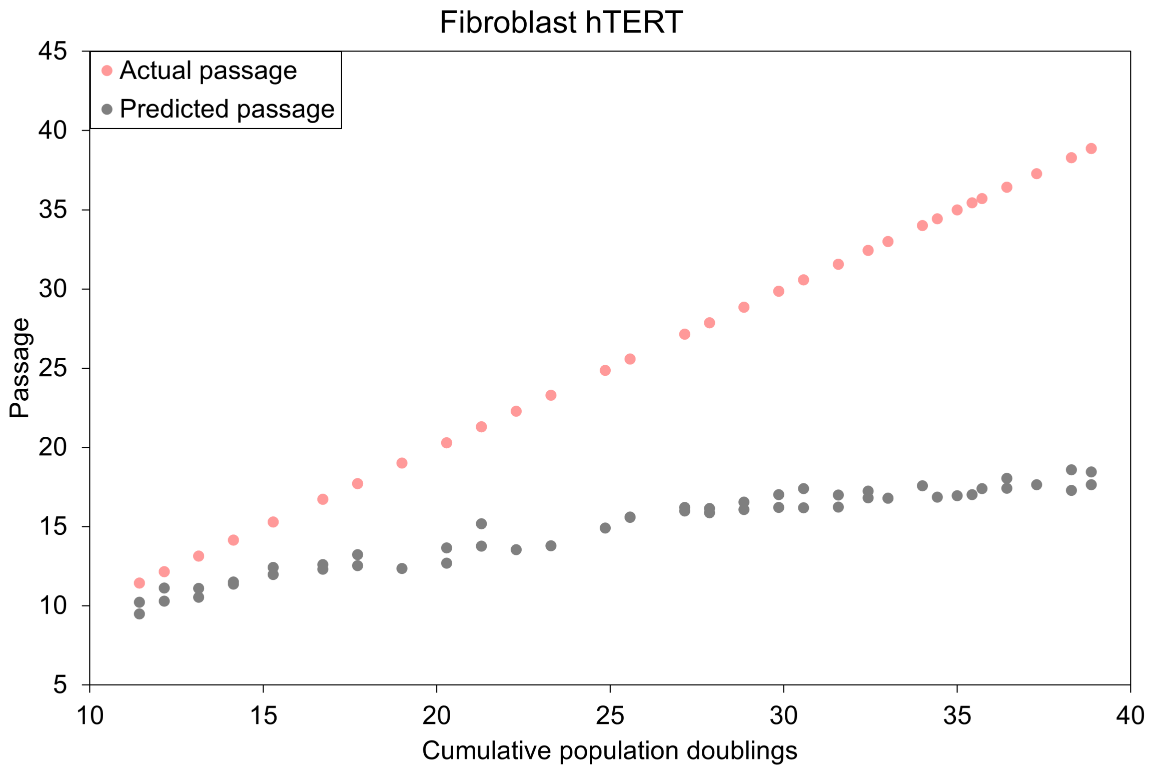

Fig. S3 A) The CellPopAge Clock predictions of Human Dermal Fibroblasts (HDF) immortalized with hTERT. Values and experimental details represented in Table S4.

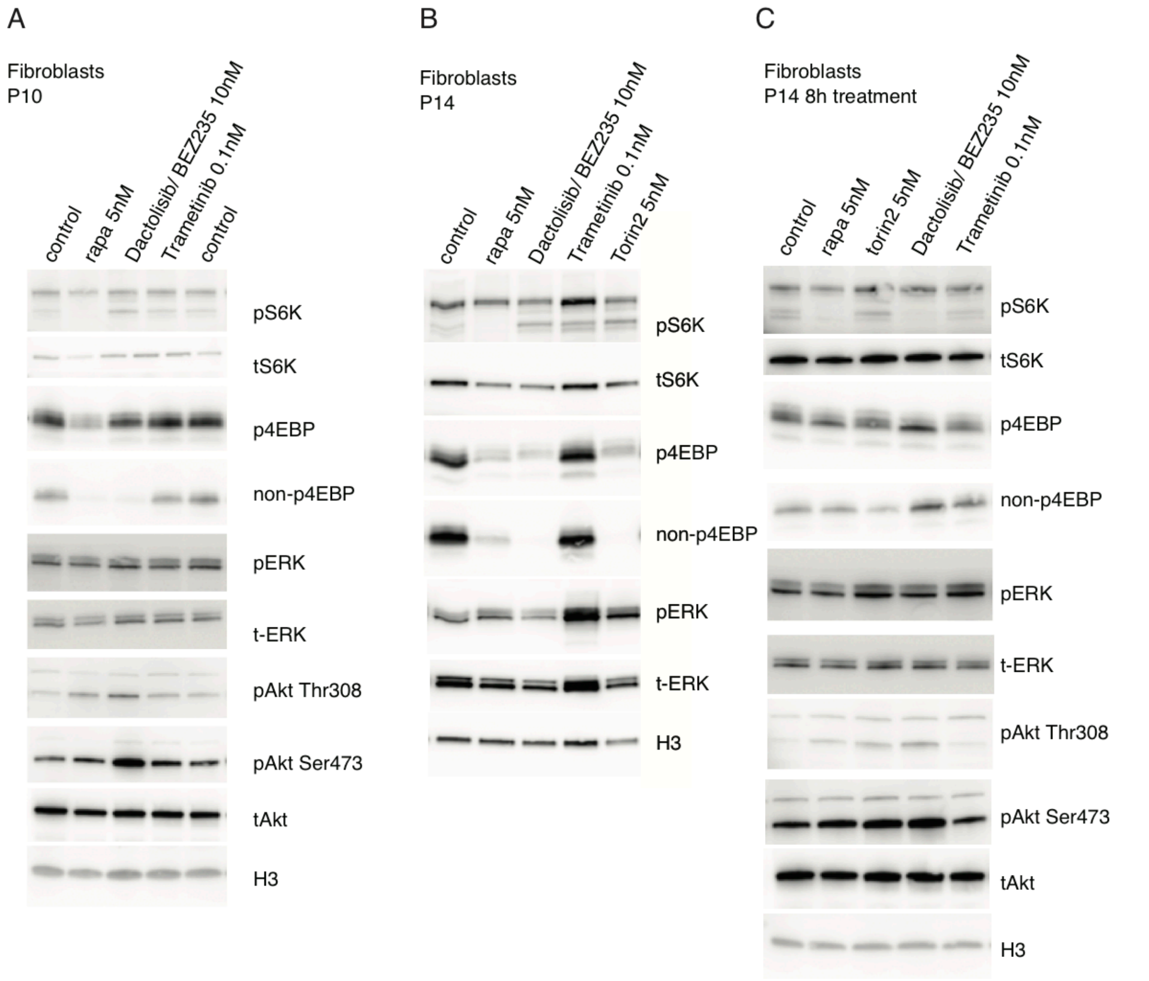

Fig. S4 Western blot analysis on HMFs treated with anti-ageing drugs.

Western blots were probed for phospho-S6K (Thr 389) showing p85 S6K Thr412 and lower band p70 S6K Thr389, total S6K, phospho-4EBP (Thr37/46), non-phospho-4EBP, pERK (Thr202/Tyr204), total ERK, and H3 as a loading control. Western blots analysis of A) HMF passage 10 and B) HMF passage 14 C) HMF passage 14 only 8h treatment with anti-ageing drugs. The analysis reflects rapid (S6K) and slower (4EBP) decreases in phosphorylation, increased phosphorylation (Akt Ser 473 and Akt Thr308) owing to S6K-IRS negative feedback loop, and resilience to phosphorylation changes owing to pMEK rebound and pERK feedback reactivation.

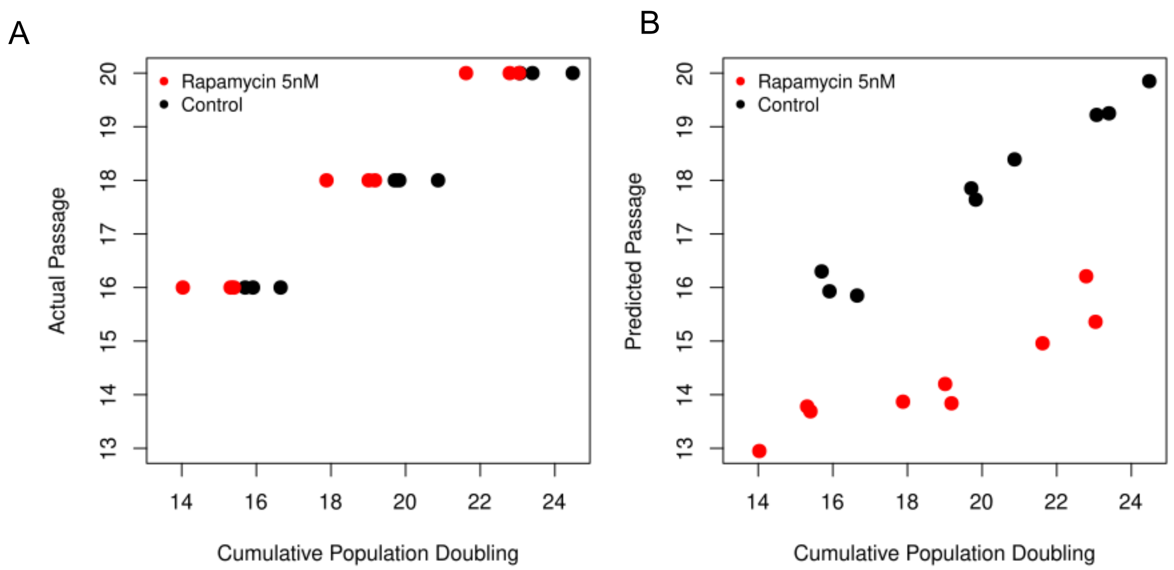

Fig. S5 Comparison of cumulative population doublings following treatment with 5nM Rapamycin and control cells that were treated with ethanol as a vehicle control.

A) A scatterplot of the cumulative population doubling against the actual passage for control and Rapamycin samples.

B) A scatterplot of the cumulative population doubling against the predicted passage from CellPopAge Clock for control and Rapamycin samples.

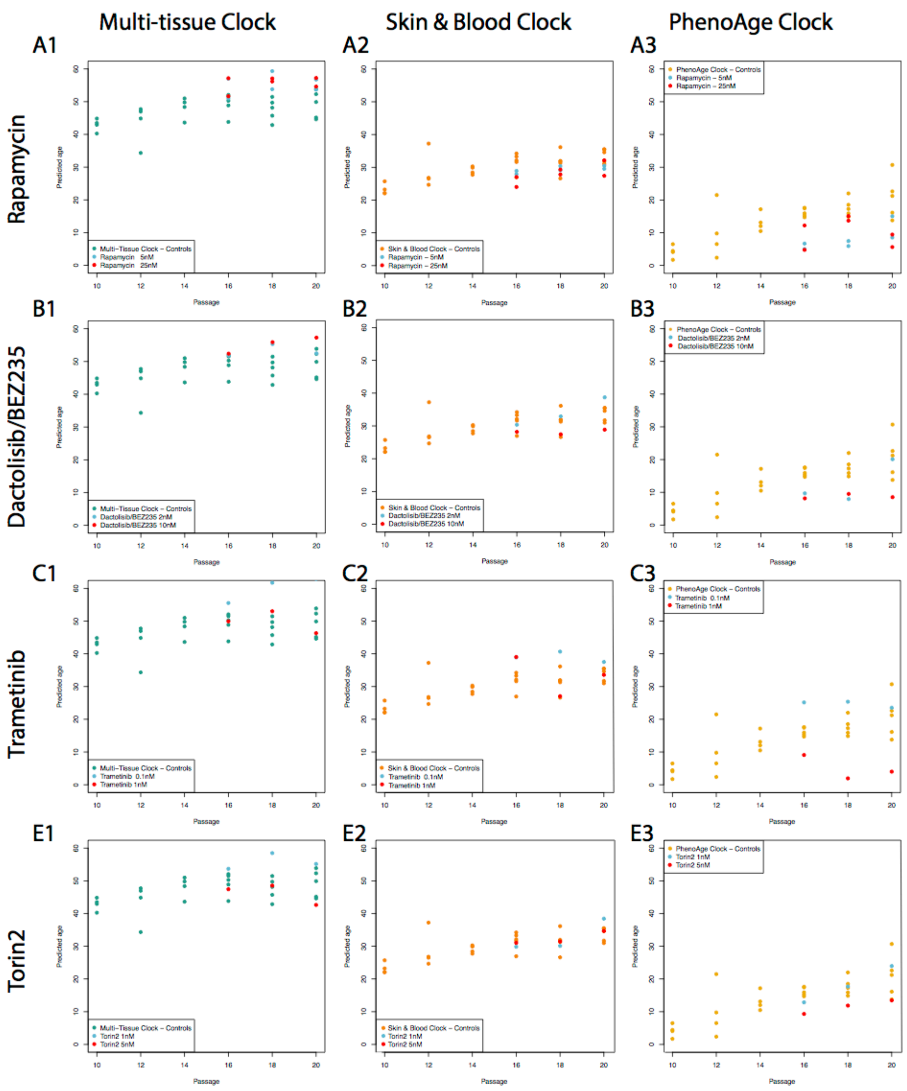

Fig. S6 Predicted age of control samples and samples treated with potential anti-ageing drugs using three existing epigenetic clocks.

Predictions of epigenetic age made using the Multi-Tissue (A1-D1, green), Skin & Blood (A2-D2, orange) and PhenoAge (A3-D3, yellow) clocks. Age predictions are indicated for samples treated with Rapamycin (A1-A3), Dactolisib/BEZ235 (B1-B3), Trametinib (C1-C3), Torin2 (D1-D3), as compared with the set of control samples. These comparisons show that the existing clocks do not consistently indicate the anti-ageing effects of these treatments on cultured cells.
