# Supplementary material for: An expedited screening platform for the discovery of anti-ageing compounds in vitro and in vivo
Source: Genome Med. 2024 Jul 2;16:85. doi: 10.1186/s13073-024-01349-w (PMC11218148; doi:10.1186/s13073-024-01349-w)
Supplement: Supplementary file 3 — Additional file 3: Table S2. Spearman's rank correlation and RMSE of the different clocks' predictions with actual cell passage of 26 samples that were not used to build the clock. [file 13073_2024_1349_MOESM3_ESM.pdf]

Supplemental Table 2: *Spearman's rank correlation and RMSE of the different clocks' predictions with actual cell passage of 26 samples that were not used to build the clock.*

Clock	Spearman’s Rho	p-value	RMSE
Multi-tissue Clock	0.41	0.03837	33.21
PhenoAge Clock	0.62	0.00070	14.01
Skin and Blood Clock	0.81	4.8e-07	11.89
CellPopAge Clock	0.98	< 2.2e-16	0.79
